# Supplementary material for: Prevalence and predictors of depressive symptoms among school-going adolescents in Kenya
Source: PLOS Ment Health. 2025 Nov 4;2(11):e0000474. doi: 10.1371/journal.pmen.0000474 (PMC12798581; doi:10.1371/journal.pmen.0000474)
Supplement: Attachment — Submitted filename: Response to Reviewers.docx [file pmen.0000474.s003.docx]

**Lucy M. Magige**

**P.O. Box 54840 00200**

**Nairobi, Kenya**

**Tel: +254716188731**

[**lmagige@kemri.go.ke**](mailto:lmagige@kemri.go.ke)

**September 29, 2025**

**PLOS Journal**

**Mental Health**

Dear Sir/Madam,

**REF: REBUTTAL LETTER RESPONDING TO EDITORS AND REVIEWER(S) COMMENTS**

I would like to appreciate the reviewers for taking time to review and offer critical insights that will strengthen the manuscript. In addition to this letter, other review concerns have been addressed in the manuscript and editor queries sorted in the submission platform.

Clarify Terminology of “Depression”
The use of “probable depression” throughout the manuscript may be misinterpreted by readers as a clinical diagnosis. As the PHQ-A is a screening, not diagnostic tool, the authors should ensure that this distinction is clear in the abstract, results, and discussion sections.

- **The terminology has been changed to depressive symptoms to reflect the screening tool and has standardized across the manuscript for clarity.**

Limitations: Generalizability
The study is limited to three sub-counties in Kakamega County. The discussion section should more explicitly acknowledge the potential limitations of generalizing the results to other geographic or socioeconomic contexts in Kenya.

- **Geographic location as a study limitation has been explicitly acknowledged under the discussion and study limitation section.**

Timing and Contextual Factors
There is limited information on the timing of data collection in relation to academic calendar events or potential psychosocial stressors. A brief mention of this context would help readers assess external influences on depressive symptoms.

- **Timing of data collection has been added in the methodology section under participant selection.**

Manuscript Language
While generally clear, some sections, particularly the discussion contain repetitive phrasing. A light edit for conciseness and clarity would improve readability.

- **Grammar checks and edits have been applied across the document to improve clarity**

Mixed Methods Mention
Although a mixed methods design is noted, this manuscript presents only quantitative results. Consider either omitting the mixed methods mention or indicating that qualitative findings will be published separately.

- **An additional line on qualitative findings being published separately has been added under the study design section.**

The statistical analyses are appropriate for the study design and objectives. The use of bi-variate and multivariable logistic regression is suitable for exploring predictors of depressive symptoms. However, the manuscript would benefit from consistent terminology (e.g., “multivariate” vs. “multiple” regression) and clearer reporting of odds ratios, confidence intervals, and cut-off points used for depression screening (particularly in the abstract and results sections). These are minor issues that can be addressed during revision and do not detract from the overall rigor of the analysis.

- **The manuscript has been revised and terminologies standardized across the manuscript. We have maintained multivariable analysis as the standard term and the odds ratios, confidence intervals have been included in the tables. Additionally cut off for depression screening(**PHQ-A ≥ 10 ) **has been included in the abstract and results sections.**

The manuscript is well-written and understandable. The English used is standard and sufficiently clear for a wide academic readership. However, there are minor issues with grammar, phrasing, and consistent use of terminology (for example, alternating between “depression,” “probable depression,” and “depressive symptoms”) that should be addressed during revision. Improving structural clarity, particularly in the Introduction and Discussion sections, will further enhance the manuscript’s readability and scholarly tone.

- **The manuscript has been revised to check for grammar, phrasing and terminologies such as probable depression has been standardized across the document.**

Abstract
Line 36: Please specify the age range of the study participants

- **The age range of the participants was 13-19 yrs and this has been included.**

Line 40: Mention the cutoff score used for PHQ-A to define probable depression.

- **A cutoff score of ≥10 on the PHQ-A was used to define probable depression and this has been included.**

Line 45: Clarify whether the regression model is multivariate or multiple. Additionally, clarify whether the authors examined correlation or association.

- **The regression model is multivariable and associations were examined**
  In the Results section, add frequency counts (n) alongside percentages where appropriate.
- **Frequency counts have been added alongside the percentages in the results section.**

Line 48: Provide the full age range (minimum and maximum values).

- **The age range has been provided (13-19yrs) in the results section**

Line 48: Rephrase the sentence “The prevalence of depressive symptoms ranged from 2.9% for moderately severe depression to 36.9% for mild depression” for greater clarity and readability.

- **The sentence has been rephrased to improve readability by maintaining the overall probable depression prevalence rate.**

  Introduction

The flow transitions abruptly from global data to Kenya-specific context. Consider restructuring into clearly defined subsections:
-Global burden and trends
-Regional context (LMICs/Sub-Saharan Africa)
-Kenya-specific findings
-Psychosocial risk factors
-Impact of COVID-19
-Research gaps and rationale

- **The introduction has been restructured to clear sub-sections for easier transition and flow.**

Present the rationale and objective in a clear, standalone paragraph.

- **The rational and objectives have been revised for clarity and as standalone paragraphs.**

Adopt active voice wherever possible for improved clarity and reader engagement.

- **Active voice has been adopted across the manuscript.**

Line 96: Clarify how school-related and family-level factors interact to influence adolescent depression.

- **The interaction between school-related and family-level factors and their influence on depression has been further elaborated in the introduction section.**

Line 124: Include examples of other countries (in addition to Kenya) where similar research has been conducted, with appropriate citations.

- **More literature on similar research conducted outside has been added including appropriate citations.**

Methodology

Line 142: Condense the geographic description of Kakamega to focus on its relevance to the study context.

- **To condense the geographic description of Kakamega and focus on relevance to study context, a map has been added**

Lines 154–155: There is inconsistency between the population described in the abstract/introduction (rural and semirural) and this section (urban and peri-urban). Please clarify and use consistent terminology.

- **The terminology has been standardized to rural and semi-rural across the document for clarity and consistency.**

Lines 159–161: Ensure consistency in referencing the study setting. Butere subcounty is mentioned in the Study Area but not the Study Setting section; this needs to be reconciled.

- **This has been reconciled to ensure all three sub-counties are highlighted**

Line 166: The total sample size is not specified here though it appears later in the Results. Please state the number of participants explicitly in this section.

- **The sample size of 1050 participants has been clarified in the sample size section.**

Line 168: Provide the parameter values used in sample size calculation, along with appropriate reference.

- **The parameter values used in sample size calculation have been added with appropriate reference**

Line 188: Clearly define "probable depression." Also, note that PHQ-A is a screening tool, not diagnostic.

- **Probable depression has been defined as a cutoff score of ≥10 on the PHQ-A. This has been clarified in the document under data sources.**

Line 191: Provide clearer descriptions of all instruments used, including the cut-off scores for identifying depressive symptoms (PHQ-A).

- **Cut off for identifying probable depression has been clarified in the data sources section**

Line 224: Clarify whether the regression model is multivariate logistic regression or multiple logistic regression.

- **The regression model is multivariable. This has been clarified in the results section.**

  Results

Include frequencies (n) along with percentages throughout the text and tables where appropriate.

- **Frequency counts have been included alongside frequencies in the result section.**

Line 236: Specify the age range of participants.

- **The age range has been added in the section.**

In Table 1:
-Rename variables for clarity.

- **The variables have been renamed.**

-A p-value is mentioned in the caption but does not appear in the table; please correct this inconsistency.

- **This has been corrected in the table.**

Line 263: The sentence does not accurately reflect the data presented in Table 2. For instance, Table 2 shows 15.8% probable depression in Malava sub-county.

- **This inconsistency has been corrected.**

Lines 262–264: Please specify the cutoff score used to define "probable depression" based on the PHQ-A.

- **Probable depression has been defined as a cutoff score of ≥10 on the PHQ-A. This has been clarified in the document under data sources.**

Lines 265–268: The odds ratio for urban population is provided, but the terminology ("urban," "semi-rural") is used inconsistently throughout the manuscript. Standardize terminology for clarity.

- **The terminology “semi-rural” has been retained and standardized across the manuscript**

Line 275: Clarify whether "Form One student" is being referred to. As written, the sentence implies that only one student had the lowest rate of depression.

- **Form one student according to the Kenyan education system meant students in the 1^st^ year of secondary school. A universal terminology has been adopted to clarify further.**

Tables 2–5: Please revise "95CI" to "95% CI" throughout.

- **This been revised.**

  Discussion

Lines 455–462: Include appropriate citations for all claims made.

- **The discussion section has been revised with appropriate citations for claims made.**

The discussion is dense and at times lacks a thematic structure. Consider:
-Synthesizing findings under key themes
-Reducing redundant or loosely connected points
-Enhancing flow with stronger transitions
Some statements are speculative; ensure these are supported with evidence or rephrase more cautiously.
Inconsistencies in terminology - “depression,” “probable depression,” “depressive symptoms” are noted. It is recommended to standardize to “probable depression” throughout for consistency and accuracy.

- **The discussion section has been revised and condensed under key themes to enhance flow and provide stronger transitions. All terminologies have also been standardized for consistency and accuracy.**

Justification for Target Age Group
The study focuses on adolescents attending secondary schools aged 13–18 years. However, it would be helpful to clarify the specific rationale for choosing this age group. Are there developmental, educational, or psychosocial factors that make this population particularly relevant for examining depression outcomes in the post-COVID-19 context?

- **A detailed justification for the target group has been provided in the background section highlighting the vulnerability to psychosocial cues and onset of mental health conditions at adolescence, and further on depression outcomes in the post-covid 19 context given the disruption of normal routine and limited social interactions a key support pillar for adolescents.**

Lack of a Stated Hypothesis
The manuscript does not clearly articulate a hypothesis. Presenting a specific, testable hypothesis would strengthen the study’s framing and help readers better understand the direction and expectations of the analysis.

**Being an exploratory study, we have incorporated the study objective in the methodology section to guide on the direction and expectations of the analysis.**

Overly Detailed Geographic Description
While the description of sub-counties and school types is informative, it is somewhat lengthy and may overwhelm readers unfamiliar with the region. Summarizing this information using a table or geographic map would enhance clarity and allow readers to more easily grasp the sampling framework.

- **A geographic map of the study area has been used to replace the lengthy description and a table used to show the sampling framework for clarity and readability.**

Missing Sampling Details
Although the methodology outlines proportional sampling by gender and form level, it does not specify the final sample size, response rate, or the handling of non-responses. These are critical for evaluating the representativeness of the sample and potential selection bias. Including these details would improve the transparency and rigor of the methodology.

- **The methodology section has now been revised to include the sample size , sampling frame and the response rate. Additionally, non -response non-response was catered for at the sample calculation step by including an attrition rate of 10%. Also , for any participant who was unavailable, replacement was done using the same selection criteria used, I.e random sampling and proportionate to gender and class allocation. This has been highlighted in the sample size calculation and participant selection section.**

Insufficient Information on Researcher-Designed Questionnaire
The study employs a researcher-designed questionnaire to assess additional psychosocial risk factors (e.g., bullying, family structure), yet no information is provided on its development, piloting, or psychometric properties. A brief description of the design process and any reliability testing would strengthen the credibility of the instrument.

- **The Methods section has been expanded to include a detailed description of the development and validation process for the researcher-designed questionnaire. The tool was developed to capture psychosocial risk factors that had been reported extensively in similar studies within the same setting. For content validity, the draft questions were reviewed by a mental health expert who is part of the investigators and then pretested and piloted in two schools separate from the sampled schools in the study county of Kakamega. Feedback from these two activities guided the revision of items to ensure clarity, cultural appropriateness, internal consistency and reliability. The refined tool was then administered in the main survey. This has been included in the data sources/measurement section.**

6. Lack of Graphical Presentation in Results
The results section relies solely on tables to present findings. While tables are useful for displaying detailed numerical data, the absence of visual aids limits the interpretability and impact of the results. Given the number of variables explored—such as gender, school type, trauma, and substance use—incorporating visual elements like box plots (to show depression score distributions across subgroups), bar charts, or forest plots (for logistic regression outcomes) would enhance reader comprehension and engagement.

- **For visuals we only considered the results for PHQ9-A,CRAFFT and CATs tools. However to show the performance and distribution of the counts and percentages of different variables from bivariate to multivariable, we utilized tables.**

The reliance on stepwise regression may inflate type I errors (false positives). The results of the regression could be contrasted with those of a cross-validation or penalization regression (e.g., LASSO) for variable selection.

- **The statistical analysis plan has been revised to highlight the fitting of LASSO to address potential multicollinearity, reduce overfitting and improve predictor stability.**

Many variables were included, which results in a more comprehensive evaluation of depression and associated symptoms and demographic factors. However, the introduction of so many variables may also introduce many correlated factors (which may over-adjust the model or introduce bias). There is no mention of a check for multicollinearity (e.g., through the VIF)

- **Multicollinearity checks were done and Tolerance values for all predictors were >0.5 and variance inflation factors (VIFs) were <2, indicating absence of multicollinearity. This has been highlighted in the statistical analysis plan.**

Similarly, there is no mention of model discrimination (e.g., AUC/ROC). Without this, there is not assessment of model performance, i.e., how well the model fits the data or how reliably it “predicts” probable depression.

- **Model discrimination has been included in the model assumptions diagnosis as “ ROC analysis shown good discriminatory ability (AUC=0.84; 95% CI: 0.81–0.87, p<0.001)”. Also, further assessment on the same is highlighted in the sensitivity analysis section.**

Once again, the risk of false positives is increased by using variables in multiple bivariate analyses. Was any multiple testing correction, e.g., Bonferroni, utilized? If not, it should be explained why.

- **We did not apply Bonferroni or other multiple testing corrections, Instead, we used a more liberal threshold (p < 0.10) at the bivariate stage to avoid prematurely excluding variables that that could have become significant after adjustment. To enhance robustness and reduce false positive, we further complemented this with LASSO penalization and bootstrap resampling, which provided a more reliable assessment of predictors stability.**

Were any sensitivity analyses conducted? For example, control analyses with alternative cut-off points or interaction terms (e.g., known interaction between age and sex, and since there were significant age effects, but apparently non-significant sex differences). Similarly, a model that only included the variables that were statistically retained by the step-wise regression (without including the ones that didn’t seem to be relevant and were “forced” into the model, e.g., the “key plausible factors”, line 220) would be interesting as a control analysis.

- **Several sensitivity analyses were done to evaluate the robustness of the final model; by using alternative PHQ9-A cut offs(≥5, ≥8, ≥10, and ≥15) , assessing potential interaction and using alternative predicted probability thresholds. Further, Bootstrap resampling (1,000 iterations) was performed to assess the predictors stability. This is elaborated in the sensitivity analyses section.**

Cultural validation of PHQ? Also, effect of cultural background on symptom reporting? (It is discussed that these topics are somewhat tabu, but there are so in many countries; further elaborating this may be beneficial to understanding).

- **The PHQ-A has been validated for use in Kenya among adolescents which means that its culturally sensitive. Additionally, the research assistants who were already trained psychologists were further trained on how to build rapport with the participants and ask the questions that cater to the cultural sensitivity. This has been elaborated in the data collection section.**

Some elaboration on concepts that may not be internationally understood, e.g.:

o Lines 237-238: “forms two to three”; what ages are these, exactly? School systems across countries vary greatly, and although the age range for this study is adolescence, it would be helpful to know what age(s) correspond to which forms.

- **Form twos and threes are basically students in the 2^nd^ and 3^rd^ year of secondary school and usually range from 15-17yrs. This has been clarified in the results section to fit into a broader understandable category.**

Also, why are there students above 18? Is this common in Kenya (i.e., is schooling 13 years long instead of the 12 years that are typical in western countries, or is it because more children repeat grades or enter school later than 6y.o.)?

- **Its common to find students who are older than 18 years in secondary schools in Kenya especially in the rural and semi-rural areas due to financial constraints which mean some start school later, repeating grades and school dropout due to lack of fees or teenage pregnancy and therefore re-entry when the age has progressed. Context around this has been also included in the manuscript**

Also in line 238, what ages were the four students that represent the smallest proportion (and how can 4 students (out of 1000) be 19%)?

- **The form fours in this case students in the 4^th^ year of secondary school are typically ages 17-18 or older and due to different socio-economic factors in Kenya, most students do not progress to higher grades such as the last year of secondary schools and therefore are usually low. This has been highlighted in the results section.**

“Harambee” in Table 1 ◊ would benefit from an explanation in the Table footing

- **A translation has been provided alongside the term to clarify it for broader readership.**

Line 404; “national examinations” (is it an exam taken at the end of high school? A short subclause within the sentence to clarify this would help)

- **Yes, national examinations meant end of secondary school examinations, this has been revised in the section.**

Table 1 would profit from some formatting; also, n=1020 is the number of variables? Or of participants? The way it’s presented here is misleading. Further, it should be indicated that the numbers in brackets (starting at “County”) are percentages.

- **The table has been formatted to make it clearer and the percentage has been denoted appropriately.**

Line 264 is missing the cut-off value, which is essential for understanding the severity of the mentioned prevalences. Specify how probable depression was defined from PHQ-A scores.

- **Probable depression has been defined as a cutoff score of ≥10 on the PHQ-9. This has been clarified in the document under data sources.**

Lines 275-277; is this a longitudinal study? How come one student was measured in the four forms? Or does this mean that the lowest prevalence per form was 9.8, 13.9, 20, 20.7%? This needs clarification because the sentence makes it seem as if it were a longitudinal study.

- **It meant that students in the first year of secondary school the lowest prevalence was 9.8% while the 4^th^ year students had the highest 20.7%. This has been clarified.**

Line 294; there’s a part of the sentence missing here.

**This has been corrected.**

- Line 341; the sentence says “after adjustment” in the beginning, but “before and after adjustment” at the end. This seems like a contradiction, which is it?

**This has been corrected.**

- Was there any missing data? How was this handled?

- **There were small amounts of item-level missing data (ie, 1013 completed CRAFFT, 1006 completed CATS). These cases were excluded listwise for the specific analyses especially in the Multivariable analysis. Given the small proportion of missingness (<2%) and most being derived variables as the ones mentioned, no imputation was applied. This has been mentioned in the statistical analysis plan section.**

Recommendation
- Many errors are present in the text, e.g., omissions of words or whole subclauses of sentences. The text must be revisited extensively, ensuring completeness and correctness. This lack of attention to detail in the text may indicate lack of thoroughness when performing the statistical analyses.

- **The entire manuscript has been revised and omissions and errors corrected to make it complete and correct.**

Many sentences and statements must be clarified, as they are not understandable or seem misleading, see comments under “Minor Concerns” above.

- **Clarifications for misunderstood or misleading statements has been done to enhance the manuscript.**

Albeit the statistical methods seem thorough for a prevalence study, some additional tests may enhance the robustness and thus credibility of the results. See the recommendations under “Major Concerns”. My fear is that the effect sizes are not stable, or confounded by the logistic regression model (since we don’t know what the model performance/quality is.

- **We addressed the stability of predictors through conducting model performance diagnostics and sensitivity analyses, including checks for multicollinearity, model fitness (omnibus test, Cox & Snell R², Nagelkerke R²), calibration (Hosmer–Lemeshow test), discrimination (ROC/AUC) and robustness checks through alternative PHQ-9 cutoffs, probability thresholds and bootstrap resampling (1,000 iterations) which demonstrated stable and credible effect sizes. This has been provided in the model performance and the sensitivity analysis sections.**

After these revisions, I recommend the paper to be accepted for submission, as it strives to fill an important gap in research regarding mental health in LMICs, which may motivate further research into culturally-dependent intervention strategies.
